# The addition of chemotherapy to radiotherapy did not reduce the rate of distant metastases in low‐risk HPV‐related oropharyngeal cancer in a real‐world setting

**DOI:** 10.1002/hed.25679

**Published:** 2019-02-04

**Authors:** Stephen F. Hall, Rebecca J. Griffiths, Brian O'Sullivan, Fei‐Fei Liu

**Affiliations:** ^1^ Department of Otolaryngology and Division of Cancer Care and Epidemiology of the Queen's Cancer Research Institute Queen's University Kingston Ontario Canada; ^2^ Cancer Care and Epidemiology at the Queen's Cancer Research Institute, Queen's University Kingston Ontario Canada; ^3^ Department of Radiation Oncology University of Toronto Toronto Ontario Canada

**Keywords:** chemoradiotherapy, distant metastases, HPV status, oropharynx, population‐based

## Abstract

**Background:**

Distant metastases (DM) are a leading cause of death for patients with oropharyngeal cancer (OPSCC). The objective of this study was to compare the rates of DM after chemoradiotherapy (CRT) and radiotherapy alone (RT) in patients with human papillomavirus (HPV)‐positive and HPV‐negative OPSCC.

**Method:**

In a retrospective population‐based study of 525 patients across Ontario, Canada, in 1998/99/03/04, we compared treatment effectiveness using cumulative incidence function curves and cause‐specific Cox regression models.

**Results:**

Sixty of 525 patients developed DM. There was no difference in rates (overall 10%‐15%) between HPV‐positive and HPV‐negative patients or between CRT‐ and RT‐treated patients. CRT reduced the risk of DM for the 15% of all HPV‐positive patients with higher risk (T4 and/or N3) and not for HPV‐negative patients (hazard ratio, 1.82 [0.65‐5.07]).

**Conclusion:**

The addition of platin‐based chemotherapy to conventional RT did not decrease the rates of DM in the majority of patients with HPV‐positive or in HPV‐negative OPSSC.

## 1 INTRODUCTION

In the early 2000s, the standard of care for many patients with squamous cell carcinoma of the head and neck, including oropharynx, evolved from radiotherapy (RT) to chemoradiotherapy (CRT) based on clinical trials (including Calais et al.,^1^ Denis et al.,^2^ Jeremic et al.,^3^ Wendt et al.^4^), meta‐analyses (Pignon et al.,^5^ Bourhis et al.^6^), systematic reviews (Browman et al.,^7^ Hao et al.^8^), and clinical practice guidelines^9^ that reported improved survival and improved local‐regional control. Subsequent meta‐analyses have supported that evidence,^10^, ^11^, ^12^ but there is little information on the impact of concurrent CRT specifically on the rate of distant metastases (DM). The only supporting evidence is a 2.5% improvement in 5‐year DM (hazard ratio [HR], 0.88; *P* = 0.04) reported by Pignon et al. in 2009^11^ that was based on 93 trials with heterogeneous patient populations, chemotherapy protocols, and RT regimens. The GORTEC trial^13^ that compared platin‐based concurrent CRT to conventional RT for oropharyngeal cancer (OPSCC) patients did not find a difference in DM. Some studies report crude proportions of DM without adjusting for competing risk and most do not have sufficient events to investigate prognostic factors. All the studies, however, predated our knowledge about and testing for human papillomavirus (HPV) leading Mroz^14^ to conclude that “the interpretation of these studies might have been misled by what we now know to have been the substantially different tumor biology of the undetermined numbers of patients with HPV+ve OPSCC.” More recently, O'Sullivan et al.^15^ found similar rates of DM comparing HPV‐positive with HPV‐negative OPSCC patients and reported that the impact of risk factors such as N Category and T Category varied between HPV‐positive and HPV‐negative patients. They suggested cautiously that lower risk patients could be candidates to withhold or reduce chemotherapy. Similarly, other authors have found no difference in the rates of DM between HPV‐positive and HPV‐negative patients^16^, ^17^ although there appears to be differences in time to DM^15^, ^16^, ^18^ and in locations of DM^18^, ^19^, ^20^ when comparing HPV‐positive with HPV‐negative patients.

DM is a leading cause of death in HPV‐positive OPSCC, yet our understanding of the true impact of the addition of chemotherapy to RT on DM for HPV‐positive or HPV‐negative OPSCC is essentially not known. The objective of this study was to compare the rate of DM in patients with OPSCC treated with concurrent CRT vs conventional RT alone in a population‐based data set of unselected real‐world patients. The study is based on data from a previously published unique observational population‐based real‐world study^21^ that compared patients, treatments, and outcomes from across the Province of Ontario, Canada, in the prechemotherapy era of 1998/99 to the initial postchemotherapy era of 2003/04.

## 2 METHOD

This is a retrospective cohort study based on 610 patients with OPSCC diagnosed between January 1,1998 and December 31, 1999 and between January 1, 2003, and December 31, 2004, who were treated for cure with either RT or CRT and on whom we could obtain tissue and HPV status via external laboratory blinded testing using either p16 or both p16 and in situ hybridization. The patient identification, chart abstraction, data collection, variables, patient data, treatment details, HPV testing protocols, HPV results, causes of death, and survival outcomes are described in previous publications.^21^, ^22^, ^23^ Staging was based on TNM sixth edition. Of the 610 patients, 243 were from the 98/99 cohort, 199 were treated with CRT and 392 were HPV‐positive. Only 3 of the 610 had known HPV status at the time of treatment. The data abstraction included up to second relapse after a disease‐free statement on the chart following initial treatment. In the absence of consistent and reliable information on smoking on the patient charts, smoking status was estimated using the Cumulative Illness Rating Scale^24^ based on information on the charts. The Cumulative Illness Rating Scale is a 13‐domain summative predictive index of comorbidity and its 5‐level respiratory domains closely align to severity of chronic obstructive pulmonary disease. “Never” smokers were recorded as 0 and patients with respiratory failure were recorded as 4. To estimate smoking levels, we reduced the 5 levels to 2 (0‐1, >1) that would reflect no or light smoking vs heavier smoking.

The study population is 525 of the 610 patients who were declared disease‐free at the end of curative initial treatment with either RT or CRT. Disease status decisions were made and recorded by the attending oncologists and/or the tumor board at routine follow‐up based on the clinical, radiological and follow‐up protocols of each cancer treatment center. Residual disease and recurrent disease were distinguished based on the presence or absence of a disease‐free status statement on the chart. Curative initial treatment for this study included the treatment of residual disease as that would be assumed to be part of initial planned treatment. Overall, 54 patients were treated with daily cisplatin, 22 with weekly cisplatin, 87 with cisplatin q3weeks, 25 with carboplatin (22 on weekly regimen), and 11 with a combination of either cisplatin or carboplatin with 5FU. Cetuximab was not used during 2003/04 in Ontario. The outcomes were site of first recurrence and any DM (first or second recurrence). The mean follow‐up time was 68.2 months from diagnosis. We report the proportions of DMs by HPV status and treatment. Logistic regression (DM yes/no) was used to investigate prognostic factors for any DM controlling for treatment, HPV status, T Category, and N Category. The baseline group was CRT, HPV‐negative, T1, and N0. Six patients who were lost to follow‐up were excluded noting that their mean follow‐up was 45.2 months. To account for the competing risks of death and first recurrence, we used Gray's test for equality of cumulative incidence functions to compare rates of DM by treatment for the HPV‐positive and HPV‐negative cohorts.^25^ We included first recurrence to the primary or neck as a competing risk as second treatment might influence rates of metastases. The comparison of Cumulative Incidence Function (CIF) curves was based on chi‐squared test and the *P‐*value was reported. Cause‐specific Cox regression models^26^, ^27^, ^28^ were generated to compare risk factors including treatment for DM. The baseline variables included age (50‐59 years), male sex, comorbidity (ACE‐27 = 0^29^), T1 Category, N0 Category, subsite = tonsil, treatment = CRT, HPV‐positive, light smoking, and low risk of DM.

Patients were grouped into high and low “risk of DM” based on combinations T and N Category as per O'Sullivan et al.^15^ They defined the high‐risk group for HPV‐positive patients as T4 and/or N3 and for the HPV‐negative patients as T3‐4 and/or N3. The risk of DM was compared in our study by group including the treatment and smoking status variables.

## 3 RESULTS

**Figure 1:**
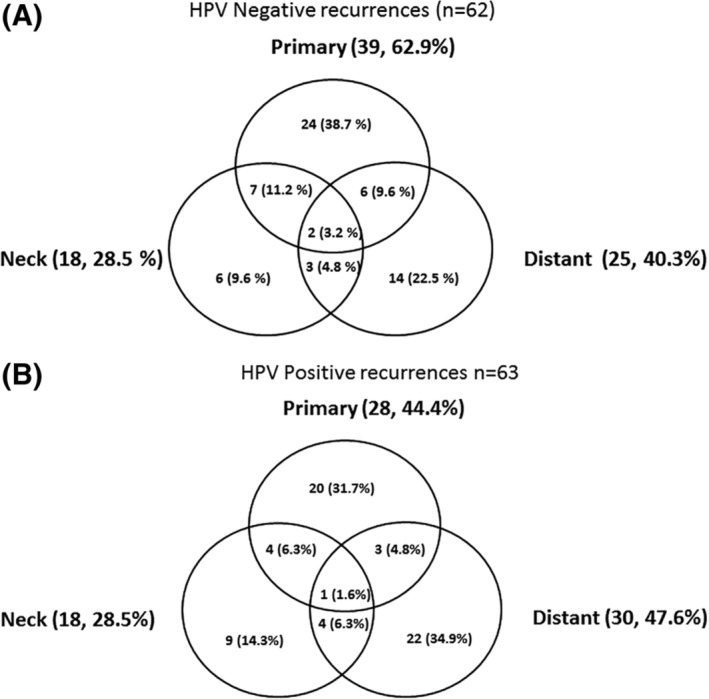
A, Sites of *first* relapse for the 62 (of the 173) HPV‐negative patients who recurred after curative treatment. B, Sites of *first* relapse for the 63 of the 352 HPV‐positive patients who recurred after curative treatment

One hundred twenty‐five of the 525 patients recurred and the sites of first recurrence are outlined on Figure 1A,B. Eventually, 60 patients developed DM, of which 36 were isolated DM.

The study population including HPV status and rates of any DM are presented in Table 1. DM occurred in 9.6% of the HPV‐positive and 15.0% of HPV‐negative patients (*P*‐value 0.07). DM occurred in 10.8% of the RT and 12.5% of the CRT patients. Overall, 200 patients died of their oropharynx, including 143 of the RT‐treated and 57 of the CRT group. One hundred eight patients died of other causes (89 RT, 19 CRT).

**Table 1:** Patient, tumor, and treatment characteristics and the rates of DM for 525 patients by HPV status

	HPV‐positive		HPV‐negative	
Variable	No. of patients	No. of patients with DM	No. of patients	No. of patients with DM
Sex				
Male	279	27	117	18
Female	73	7	56	8
Smoking				
Light	218	20	37	5
Heavy	134	14	136	21
T Category				
T1	102	6	29	4
T2	148	14	83	10
T3	64	8	33	7
T4	38	6	28	5
N Category				
N0	64	8	76	5
N1	65	2	34	5
N2	209	21	62	16
N3	14	3	1	0
Subsite				
Base of tongue	116	12	49	11
Tonsil/palate	236	22	124	15
Risk group				
High	52	9	62	12
Low	300	25	111	14
Treatment				
RT	215	19	140	20
CRT	137	15	33	6
Total	352	34	173	26

Abbreviations: CRT, chemoradiotherapy; DM, distant metastases; RT, radiotherapy alone.

The risk factors for the development of DM based on all patients using logistic regression were N3 (OR 5.03 [1.14‐22.23]) and HPV‐negative status (OR = 1.94 [1.07‐3.52]) (data not included). The addition of chemotherapy did not have a statistically significant impact on the risk of DM (OR 1.06 (0.57‐1.98) over RT alone. Similarly for the HPV‐positive cohort only, the addition of chemotherapy to RT alone did not have a significant impact (OR 0.89 [0.41‐1.92]) over RT alone similar to the results for the HPV‐negative cohort (OR 1.37 [0.45‐4.15]).

**Figure 2:**
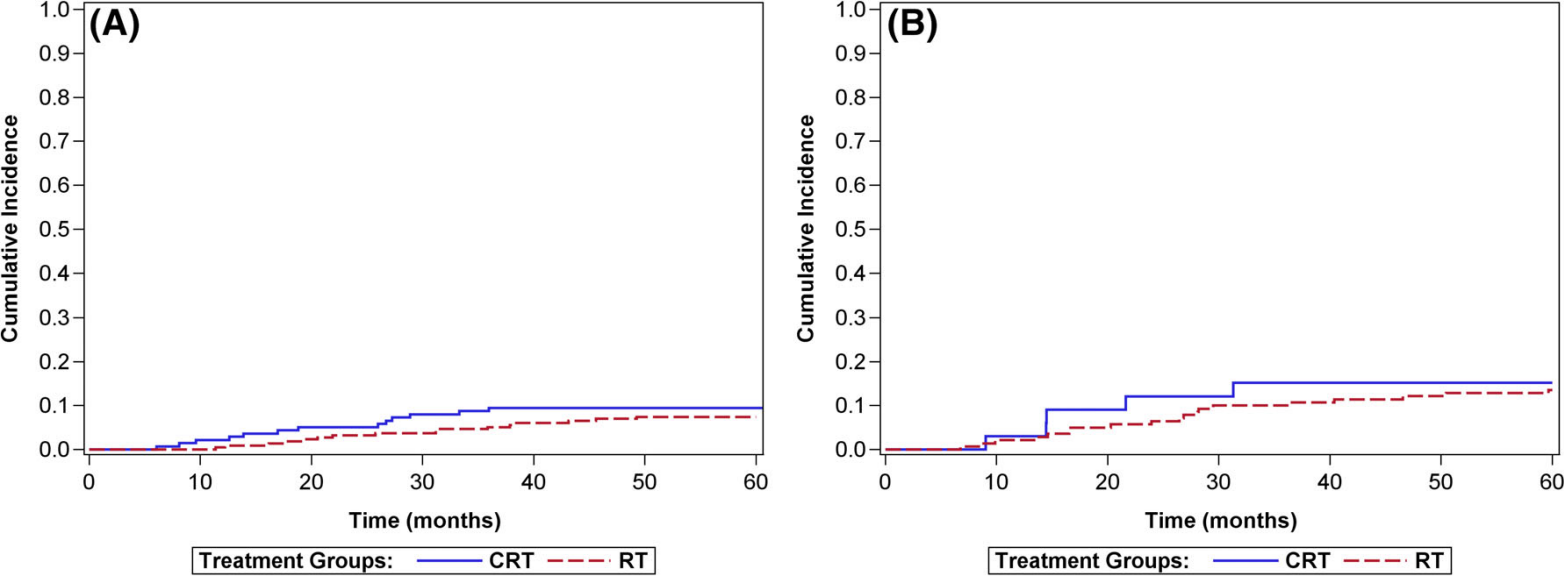
A, Cumulative incidence function curve for distant metastasis by treatment for 352 HPV‐positive patients. B, Cumulative incidence of distant metastasis by treatment for 173 HPV‐negative patients. (RT = radiotherapy alone, CRT = chemoradiotherapy) [Color figure can be viewed at wileyonlinelibrary.com]

The CIF curves comparing CRT to RT for the HPV‐positive and HPV‐negative patients are presented in Figure 2A,B. There was no statistically significant difference in rates of DM with the use of CRT for either cohort (*P* = 0.34, *P* = 0.77). Similar results were obtained when the CIF curves only accounted for death as a competing risk (*P* = 0.41, *P* = 0.85).

The results of the cause‐specific hazards model for all patients are presented in Table 2. Age, sex, comorbidity, and subsite were not significant and are not included in the table. The risk factors for the development of DM were T3, N2, N3, and HPV‐negative status. The addition of CRT was not significant (HR, 1.15 [0.64‐2.07]). When the analysis was performed for the HPV‐positive cohort (Table 3), the only statistically significant factor was T4 (HR, 4.12 [1.17‐14.57]) with only a trend toward significance in the very small number of N3 cases in the study (*n* = 17). For the HPV‐negative patients, the risk factor was N2 (HR, 6.78 [2.197‐20.94]) and CRT had no statistically significant impact (HR, 1.82 [0.65‐5.07]). Treatment, that is, CRT, did not influence the rates of DM for the HPV‐positive and HPV‐negative patients. Similar results were obtained when accounting for death only as a competing risk in the models.

**Table 2:** The hazards ratios from the cause‐specific hazard model for distant metastases based on all patients

	Hazards ratio	Lower CI	Upper CI	*P*
T Category				
T1	1.00			
T2	1.784	0.787	4.044	.1659
T3	2.898	1.169	7.179	.0216
T4	2.469	0.929	6.560	.0698
N Category				
N0	1.00			
N1	1.030	0.368	2.885	.9544
N2	2.651	1.248	5.630	.0112
N3	7.949	1.989	31.776	.0034
Treatment				
CRT	1.00			
RT	**1.152**	**0.641**	**2.070**	**.6363**
HPV status				
Negative	1.00			
Positive	0.357	0.197	0.647	.0007

Abbreviations: CRT, chemoradiotherapy; RT, radiotherapy alone.

**Table 3:** The hazards ratio from the cause‐specific hazard model for distant metastases based on the HPV‐positive patients

Variable	Hazards ratio	Lower CI	Upper CI	*P*
T Category				
T1	1.0			
T2	1.852	0.644	5.327	.2530
T3	2.402	0.696	8.298	.1658
T4	4.121	1.166	14.567	.0280
N Category				
N0	1.0			
N1	0.191	0.023	1.613	.1285
N2	0.993	0.387	2.551	.9891
N3	3.654	0.833	16.034	.0859
Treatment				
CRT	1.0			
RT	**0.894**	**0.423**	**1.891**	**.7697**
Smoking				
Light	1.0			
Heavy	1.107	0.520	2.355	.7926

Abbreviations: CI, confidence interval; CRT, chemoradiotherapy; RT, radiotherapy alone.

Table 4 presents the model for high and low “risk of DM” groups for the HPV‐positive cohort. The high‐risk group did develop more DM (HR = 2.80, 1.27‐6.17, *P* = 0.01). Smoking status was not a statistically significant factor. The model for the HPV‐negative cohort showed similar nonstatistically significant results for treatment and smoking, and high “risk of DM” group was not statistically significantly different (HR = 1.55, 0.69‐3.5).

**Table 4:** The hazard ratios from the cause‐specific hazard model for the risk of distant metastases including the variable “distant metastases risk group” and based on the HPV‐positive patients

Variable	Hazards ratio	Upper CI	Lower CI	*P*
Treatment				
CRT	1.00			
RT	0.826	0.399	1.709	.6058
Smoking				
Light	1.00			
Heavy	1.118	0.529	2.360	.7702
Risk group				
Low	1.00			
High	2.800	1.271	6.171	.0106

Abbreviations: CI, confidence interval; CRT, chemoradiotherapy; RT, radiotherapy alone.

## 4 DISCUSSION

The objective of this study was to determine, in the absence of evidence, the impact of the addition of concurrent chemotherapy to conventional RT for patients with HPV‐positive and HPV‐negative patients with OPSCC *specifically* on the rates of DM. Based on 525 patients who were declared disease‐free after curative treatment and 60 developed DM. Between 10% and 15% of patients developed DM regardless of HPV status and treatment. CRT did not influence the rate overall, for the HPV‐positive cohort or for the HPV‐negative cohort. In the most recent meta‐analysis comparing CRT to RT alone, Blanchard et al.^10^ reported a 5‐year benefit in overall survival of 8.1% (HR, 7.8) for OPSCC patients and given this advantage compared to rates of toxicity and cost, the lack of impact on DM should be of interest to patients, oncologists, funders and policy makers. The decision use CRT for any patient needs to consider all the evidence including overall survival, local control, regional control, DM, and toxicity.

Our results are consistent with those of O'Sullivan^15^ who reported no difference in the rates of DM between HPV‐positive and HPV‐negative OPSCC. They identified different risk strata for development of DM based on combinations of T and N Category for each HPV status group. Our results support their risk strata for the HPV‐positive patients as patients with T4 and/or N3 disease had fewer DM events and strongly endorse their conclusion “that lower risk patients could be candidates to withhold or reduce chemotherapy” noting that this group only represents 52/352 (14.7%) of all HPV‐positive cases. For the HPV‐negative patients, we found that overall the risk of DM was greater than the HPV‐positive patients (Table 2), that the addition of CRT did not reduce the rates of DM overall and we could not identify a higher “risk of DM” group.

The strengths of this study are the inclusion of the complete cohort of “real‐world” patients from all treatment centers in Ontario, Canada, in a time when oncologists were HPV naive. For this study we used competing risk analysis. Other strengths include the unique design that used similar patients from different eras with different rates of HPV‐positive, the systematic data collection and quality of the HPV tissue testing. Testing was done at one independent laboratory (the Molecular Oncology Lab, the Princess Margaret Hospital, Toronto, Canada) that was blind to patient identifiers, treatments, and results.

The limitations of the overall study are carefully described in the original publications.^21^, ^22^, ^23^ This is not the analysis of a clinical trial but is a meticulously performed retrospective population‐based observational study. Booth et al.^30^, ^31^, ^32^ have reviewed the “pros and cons” of RCTs compared to population‐based studies and concluded that “well‐designed population‐based outcome studies should be considered a natural step in the evolution of evidence and should be conducted in follow‐up of all major randomized controlled trials… due to the fundamental difference between efficacy and effectiveness inherent in the patient selection bias, treatment bias and settings of RCTs that differ from practice in the community.” Studies such as ours can be used to support or question controversial or confusing findings. There is no other study that compares DM in HPV‐positive and HPV‐negative patients comparing conventional RT alone to CRT.

There are six potential limitations for this substudy. First, we unfortunately did not abstract the location of the DM. This would be of particular interest, given reports of differences in DM sites.^18^, ^19^, ^20^ Second, it is possible that our results reflect differences in the effectiveness of the various chemotherapy regimens used at the time; however, there is no evidence to support this hypothesis either for survival or specifically for DM. Third, in this study of real‐world patients it is possible that differences in chemotherapy protocol completion might have affected rates of DM. There is no past evidence suggesting that reducing the total dose of chemotherapy alters the risk of DM and we have not accounted for treatment completion in the analysis. Fourth, as this study is based on treatments and salvage treatments in 1998‐2004, it is possible that we have underestimated the current rates of DM as presently more patients might survive salvage treatment only to develop DM. Differences in treatment effectiveness and imaging quality, however, would affect both the HPV‐positive and HPV‐negative cohorts and both treatment cohorts. Differences in imaging in 1998/98 are irrelevant as DM would eventually appear and there is no evidence of a curative treatment for DM in this condition. Fifth, we did not consider time to DM. O'Sullivan,^15^ Huang,^16^ and Trosmam^18^ all reported that HPV‐negative patients develop DM sooner than HPV‐positive patients and our Figure 2A,B are consistent with the findings of those authors. Finally, we did not find a relationship between smoking and DM as previously reported.^15^, ^33^ We used a surrogate for smoking based on patient chart information and thus may have underestimated smoking status effect.

## 5 CONCLUSION

Based on 525 patients with OPSCC treated for cure with either RT or CRT who were declared disease‐free at the end of treatment, we found no overall difference in the rates of DM comparing CRT with RT alone for HPV‐positive or HPV‐negative patients. HPV+ patients with the most advanced disease might benefit, but 90% of OPSCC patients regardless of HPV status need to be informed that there is no evidence for a reduction in DM with addition of platin‐based concurrent chemotherapy to conventional RT.

## CONFLICTS OF INTEREST

The authors have no conflicts of interest to report.

## AUTHOR CONTRIBUTIONS

Dr Hall and Ms Griffiths were involved throughout the study design, data analysis, manuscript writing and manuscript revisions. Dr Hall had full access to all the clinical data in the study and takes responsibility for the integrity of the clinical data and the accuracy of the data analysis.
